# Development of Benzobisoxazole-Based Novel Conjugated Polymers for Organic Thin-Film Transistors

**DOI:** 10.3390/polym15051156

**Published:** 2023-02-24

**Authors:** WonJo Jeong, Kyumin Lee, Jaeyoung Jang, In Hwan Jung

**Affiliations:** 1 Human-Tech Convergence Program, Department of Organic and Nano Engineering, Hanyang University, 222 Wangsimni-ro, Seongdong-gu, Seoul 04763, Republic of Korea; 2 Department of Energy Engineering, Hanyang University, Seoul 04763, Republic of Korea

**Keywords:** benzo[1,2-d:4,5-d′]bis(oxazole), conjugated polymers, organic thin film transistors, thiophene spacers, hole mobility

## Abstract

Benzo[1,2-d:4,5-d′]bis(oxazole) (BBO) is a heterocyclic aromatic ring composed of one benzene ring and two oxazole rings, which has unique advantages on the facile synthesis without any column chromatography purification, high solubility on the common organic solvents and planar fused aromatic ring structure. However, BBO conjugated building block has rarely been used to develop conjugated polymers for organic thin film transistors (OTFTs). Three BBO-based monomers, BBO without π-spacer, BBO with non-alkylated thiophene π-spacer and BBO with alkylated thiophene π-spacer, were newly synthesized and they were copolymerized with a strong electron-donating cyclopentadithiophene conjugated building block to give three p-type BBO-based polymers. The polymer containing non-alkylated thiophene π-spacer showed the highest hole mobility of 2.2 × 10^−2^ cm^2^ V^−1^ s^−1^, which was 100 times higher than the other polymers. From the 2D grazing incidence X-ray diffraction data and simulated polymeric structures, we found that the intercalation of alkyl side chains on the polymer backbones was crucial to determine the intermolecular ordering in the film states, and the introduction of non-alkylated thiophene π-spacer to polymer backbone was the most effective to promote the intercalation of alkyl side chains in the film states and hole mobility in the devices.

## 1. Introduction

Since the discovery of conjugated polymers that can deliver holes and electrons in a polymer chain [1,2], conjugated polymers have been actively studied for utilization in printable electronics that can realize flexible, lightweight, and robust electronic devices. Currently, tremendous conjugated polymers were developed and successfully utilized as active materials for organic light-emitting diodes (OLEDs), organic photovoltaics (OPVs), organic photodetectors (OPDs), and organic thin film transistors (OTFTs) [3,4,5,6,7,8,9,10,11,12].

In the development of conjugated polymers, the most powerful strategy is to synthesize conjugated building blocks [13,14,15], such as benzothiadiazole/benzotriazole [16,17,18,19], diketopyrrolopyrrole (DPP) [20,21,22,23], cyclopentadithiophene (CPDT) [24,25,26] and naphthalene diimide (NDI) [27,28,29]. Since each conjugated building block possesses different electron donating (or accepting) properties as well as different molecular ordering behaviors, combinations of two or more conjugated building blocks can create a variety of novel conjugated polymers that exhibit different absorptions, energy levels, and molecular ordering. Thus, finding new conjugated building blocks is the key to developing conjugated polymers with diverse electrical properties.

Benzo[1,2-d:4,5-d′]bis(oxazole) (BBO) [30,31] is a heterocyclic aromatic ring composed of one benzene ring and two oxazole rings, which has unique advantages on the facile synthesis without any column chromatography purification, high solubility on the common organic solvents and planar fused aromatic ring structure [32]. The BBO conjugated building block was first utilized in OLEDs. Jeffries-El et al. reported electroluminescence polymers composed of 9,9-dioctylfluorene and BBO building blocks and showed blue emission with luminous efficiencies of 1 Cd/A at 470 nm [33]. After that, they modified the BBO polymer structure by adding thiophene rings and showed blue-green emission with luminous efficiencies of 0.86 Cd/A at 505 nm [34]. For OPV applications, BBO moiety was copolymerized with quaterthiophene and the resulting polymers showed a power conversion efficiency of up to 1.14% under AM 1.5 solar irradiation [35]. However, BBO-based polymers have not been actively studied for a long time, but recently, our research groups have constructed novel BBO polymers by connecting polymer chains in the 4,8-direction of the BBO building block rather than its 2,6-position. Alkylated BBO building block was copolymerized with a benzo [1,2-b:4,5-b′] dithiophene building block, and we first reported a promising OPD performance with a responsitivty of 0.385 A/W and a specific detectivity of 1.13 × 10^13^ Jones at −1 V in the p-n junction photodiodes [32]. Despite successful utilization in electronic devices, BBO building blocks have not been widely used in various electronic devices.

**Scheme 1:**
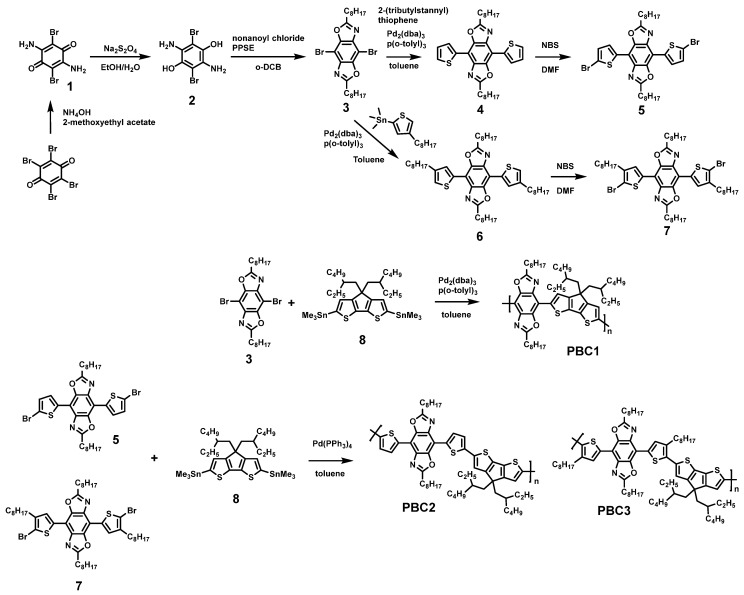
Synthetic route for PBC1, PBC2, and PBC3.

In this study, we first utilized BBO building blocks to build conjugated polymers for OTFT application. Alkylated BBO moieties were copolymerized with an electron-donating CPDT building block to increase the p-type characteristics of BBO-based polymers. In addition, two types of thiophene π-spacers (alkylated or non-alkylated) were introduced in the BBO-based polymer backbones to promote hole transporting characteristics of the polymers. Finally, Three BBO monomers without π-spacer, with non-alkylated thiophene π-spacer, and with alkylated thiophene π-spacer were copolymerized with the CPDT monomer, which produced three novel conjugated polymers, PBC1, PBC2, and PBC3, respectively, as shown in Scheme 1.

The thiophene π-spacers were significantly affected by the molecular ordering and hole-transporting characteristics of the BBO-based polymers. The BBO-based polymer containing non-alkylated thiophene π-spacer (PBC2) showed strong crystallinity in the film states whereas the BBO-based polymer having alkylated thiophene π-spacer (PBC3) did not show crystallinity in the film state. As a result, the PBC2 polymer showed 100 times higher hole mobility (2.2 × 10^−2^ cm^2^·V^−1^·s^−1^) than the PBC3 polymer (5.0 × 10^−4^ cm^2^·V^−1^·s^−1^). In the case of BBO-based polymer without π-spacer (PBC1), it showed a moderate hole mobility of 9.2 × 10^−4^ cm^2^·V^−1^·s^−1^. We found that the intercalation of alkyl side chains on the polymer backbones was important to increase the molecular ordering in the film states and hole mobility in the devices, and the introduction of non-alkylated thiophene π-spacer to polymer backbone was the best to promote the intercalation of alkyl side chains and polymer crystallinity by increasing vacant cites between BBO and CPDT building blocks.

## 2. Materials and Methods

### 2.1. Synthesis of Monomers

4,8-dibromo-2,6-dioctylbenzo[1,2-d:4,5-d′]bis(oxazole) (BBO, 3): Poly (trimethylsilyl phosphate) (13.0 g) and nonanoyl chloride (10.0 g, 44.64 mmol) were dissolved in *o*-dichlorobenzene (*o*-DCB) (50 mL). The solution was then degassed by bubbling argon through it for 30 min and then added the solution in an N_2_ atmosphere flask containing compound **2** (5.54 g, 18.6 mmol). Then the mixture was heated to 90 °C for 72 h. The solution is concentrated by vacuum distillation of the *o*-DCB. The resulting crude product was purified by column chromatography. The resulting white solid was recrystallized using dichloromethane/methanol to give compound **3** (6.37 g, 63.1%) ^1^H NMR (400 MHz, CDCl_3_) δ: 3.010 (m, 4H), 1.924 (dt, 4H), 1.444–1.276 (m, 20H), 0.879 (m, 6H).

2,6-dioctyl-4,8-di(thiophen-2-yl)benzo[1,2-d:4,5-d′]bis(oxazole) (4): Compound **3** (1.0 g, 1.84 mmol) and Pd_2_ (dba)_3_ (50.5 mg, 5.5 × 10^−2^ mmol), p-(o-tolyl)_3_ (56.0 mg, 1.8 × 10^−1^ mmol) were dissolve in toluene (28.0 mL). The solution was then degassed by bubbling nitrogen through it for 30 min and then added the solution in an N_2_ atmosphere flask containing 2-(tributylstannyl)thiophene (1.5 g, 4.05 mmol). The reaction mixture was heated to 120 °C overnight and then it was then extracted with dichloromethane and water. The crude product was purified by filtration on silica gel and then recrystallized to afford compound **4** as a luminous solid (0.600 g, 55.0%). ^1^H NMR (400 MHz, CDCl_3_) δ: 8.146 (s, 2H), 7.099 (s, 2H) 3.090 (t, 4H), 2.730 (t, 4H), 2.001 (m, 4H), 1.721 (m, 4H), 1.534–1.297 (m, 40H), 0.884 (t, 12H).

4,8-bis(5-bromothiophen-2-yl)-2,6-dioctylbenzo[1,2-d:4,5-d′]bis(oxazole) (5): Compound **4** (0.43 g, 0.5561 mmol) was dissolved in dimethylformamide (DMF) (30 mL) and chloroform (10 mL), and then *N*-bromosuccinimide (NBS) (0.247 g, 1.390 mmol) was added to the reaction mixture. After it was stirred at room temperature overnight, it was extracted with dichloromethane and water. After removing the solvent, the crude product was purified by filtration on the silica gel. The final monomer 5 was obtained from the recrystallization as a yellow solid. (0.417 g, 80.54%) ^1^H NMR (600 MHz, CDCl_3_) δ: 7.964 (s, 2H), 3.079 (t, 4H), 2.668 (t, 4H), 2.004 (m, 4H), 1.680 (m, 4H), 1.522–1.294 (40H), 0.88 (d, 12H).

2,6-dioctyl-4,8-bis(4-octylthiophen-2-yl)benzo[1,2-d:4,5-d′]bis(oxazole) (6): Compound **3** (1.528 g, 2.8181 mmol) and Pd_2_ (dba)_3_ (77 mg, 8.454 × 10^−2^ mmol), p-(o-tolyl)_3_ (86 mg, 0.2818 mmol) were dissolved in toluene (33 mL). The solution was then degassed by bubbling nitrogen through it for 30 min, and then trimethyl (4-octylthiophen-2-yl)stannane (2.4291 g, 6.7634 mmol) was added to the reaction mixture. After refluxing at 120 °C overnight, the reaction mixture was extracted with dichloromethane and water, and then dried over MgSO_4_. The resulting crude product was purified by column chromatography (dichloromethane: hexanes = 1:1 *v*/*v*%) to give compound **6** as a yellow solid (1.819 g, 83.48%). ^1^H NMR (400 MHz, CDCl_3_) δ: 8.146 (s, 2H), 7.099 (s, 2H) 3.090 (t, 4H), 2.730 (t, 4H), 2.001 (m, 4H), 1.721 (m, 4H), 1.534–1.297 (m, 40H), 0.884 (t, 12H).

4,8-bis(5-bromo-4-octylthiophen-2-yl)-2,6-dioctylbenzo[1,2-d:4,5-d′]bis(oxazole) (7): Compound **6** (0.43 g, 0.5561 mmol) was dissolved in DMF (30 mL). After adding NBS (0.247 g, 1.390 mmol) to the reaction mixture, it was stirred at room temperature overnight. The reaction mixture was extracted with dichloromethane and water and then dried over MgSO_4_. The crude product was purified by column chromatography (dichloromethane: hexanes = 1:1 *v*/*v*%) to give compound **7** as a yellow solid. (0.417 g, 80.54%) ^1^H NMR (600 MHz, CDCl_3_) δ: 7.964 (s, 2H), 3.079 (t, 4H), 2.668 (t, 4H), 2.004 (m, 4H), 1.680 (m, 4H), 1.522–1.294 (40H), 0.88 (d, 12H).

### 2.2. Synthesis of Polymers

Poly[(2,6-dioctylbenzo[1,2-d:4,5-d′]bis(oxazole)-4,8-diyl)-alt-(4,4-bis(2-ethylhexyl)-4H-cyclopenta [2,1-b:3,4-b′]dithiophene-2,6-diyl)] (PBC1)

Compound **3** (252 mg, 0.4646 mmol), compound **6** (420 mg, 0.4646 mg), Pd_2_ (dba)_3_ (12.8 mg, 1.393 × 10^−2^ mmol), and p-(o-tolyl)_3_ (14 mg, 4.646 × 10^−2^ mmol) were dissolved in degassed anhydrous toluene (9.2 mL). After polymerization, the polymer fibers were washed by Soxhlet extraction with methanol, acetone, and chloroform. The polymer solution was filtered through celite to remove the metal catalyst and the final polymer was obtained after reprecipitation in the methanol (330.0 mg, 73.87%).

Poly[(4,8-di(thiophen-2-yl)-2,6-dioctylbenzo[1,2-d:4,5-d′]bis(oxazole)-5,5′-diyl)-alt-(4,4-bis(2-ethylhexyl)-4H-cyclopenta[2,1-b:3,4-b′]dithiophene-2,6-diyl)] (PBC2)

PBC2 was synthesized by a similar procedure with PBC1 using Pd (PPh_3_)_4_ instead of Pd_2_ (dba)_3_/p-(o-tolyl)_3_. Compound **5** (200 mg, 0.283 mmol), compound **8** (206.1 mg, 0.283 mmol), Pd (PPh_3_)_4_ (16.4 mg, 1.4 × 10^−2^ mmol), and toluene (5.7 mL) were used for the polymerization. Yield: 231.0 mg (86.0%).

Poly[(4,8-di(4-octylthiophen-2-yl)-2,6-dioctylbenzo[1,2-d:4,5-d′]bis(oxazole)-5,5′-diyl)-alt-(4,4-bis(2-ethylhexyl)-4H-cyclopenta[2,1-b:3,4-b′]dithiophene-2,6-diyl)] (PBC3).

PBC3 was synthesized by the identical procedure to PBC2. Compound **7** (300 mg, 0.3222 mmol), compound **8** (234.7 mg, 0.3222 mmol), Pd (PPh_3_)_4_ (18.6 mg, 1.61 × 10^−2^ mmol), and toluene (6.4 mL) were used for the polymerization. Yield: 277.0 mg (73.2%).

### 2.3. Characterization of the Synthesized Materials

The absorption spectrum of the active layer was measured using a JASCO V-730 UV/VIS spectrometer, ^1^H nuclear magnetic resonance (NMR) spectra were recorded at 25 °C using a VNMRS 600 MHz spectrometer (California, USA), Cyclic voltammetry measurements were performed at a scan rate of 20 mV/s using a WonATech potentiostat/galvanostat/impedance analyzer ZIVE SP1 (1A) (Seoul, Republic of Korea), with a three-electrode cell and a 0.1 N Bu_4_NBF_4_ solution in acetonitrile as the electrolyte, and the working electrode was coated with the polymer films by dipping them into their solutions in chloroform. Gel permeation chromatography measurements were recorded at 35 °C using an Agilent 1260 Infinity II GPC (California, USA).

### 2.4. Device Fabrication and Characterization

To investigate the electrical properties of the PBC1, PBC2, and PBC3 thin films, OTFTs were fabricated using the top contact/bottom gate configuration. Heavily n-doped Si wafers covered with thermally grown 300 nm thick SiO_2_ dielectric layers were used as gate substrates; they were cleaned via piranha treatment, followed by repeated rinsing and sonication with deionized water. To further hydrophilize the as-cleaned SiO_2_ surface, an ultraviolet (UV)/ozone treatment was performed for 20 min in a UV/ozone cleaner (PSD PRO-UV, Novascan Tech., Chicago, IL, USA). The substrates were then immersed in an octadecyltrichlorosilane (ODTS) solution (1 vol% in anhydrous toluene) for 1 h in ambient air. PBC1–3 polymer solutions (10 mg mL^–1^ in chloroform) were spin-coated at 2000 rpm for 45 s on the ODTS-treated Si/SiO_2_ substrates. These solutions were prepared and coated onto the substrates in a glove box filled with N_2_ gas (O_2_ and H_2_O < 5 ppm). Some of the polymer films were thermally annealed for 10 min on a hot plate at various temperatures and cooled slowly to room temperature in a glove box. Next, a 100 nm-thick Au source and drain electrodes were deposited onto the active layers via thermal evaporation using a shadow mask (channel length (*L*) and width (*W*) were 100 and 300 μm, respectively).

The electrical properties of the TFTs were characterized using a Keithley 4200 semiconductor parameter analyzer in an N_2_-filled glove box. The field-effect mobility (*μ*) was extracted from the transfer curve in the saturation using the following equation: *I*_D_ = *μC*_i_ (*W*/2*L*) (*V*_G_ − *V*_th_)^2^, where *I*_D_, *C*_i_, *V*_G_, and *V*_th_ denote the drain current, capacitance per unit area (300 nm: 10.0 nF cm^–2^), gate voltage, and threshold voltage, respectively. Two-dimensional grazing incidence X-ray diffraction (2D-GIXD) measurements were performed at the 3C beamline of the Pohang Accelerator Laboratory (Pohang, Republic of Korea). A high-resolution synchrotron X-ray beam source (beam energy = 9.8 keV) with a grazing incidence angle of approximately 0.15° was used to perform the 2D-GIXD experiments. To examine the surface morphologies of the polymer thin films, atomic force microscopy (AFM) analysis was performed using an atomic force microscope (XE-100, Park Systems, Suwon, Republic of Korea).

## 3. Results and Discussion

### 3.1. Synthesis and Characterization of the Polymers

BBO compound 3 was synthesized from the previously reported synthetic procedures [32]. Compound **1** was synthesized via amination using NH_4_OH as an amine source, and then it was reduced to aromatized enol compound 2 by using a reducing agent of Na_2_S_2_O_4_. Due to the insolubility and air sensitivity of compound 2, it was directly reacted with nonanoyl chloride in the presence of poly (trimethylsilyl phosphate) support to obtain soluble BBO monomer 3. Compound 4 and 6 were synthesized by Stille coupling of compound 3 with 2-(tributylstannyl) thiophene and trimethyl (4-octylthiophen-2-yl) stannane, respectively, and then they were reacted by bromination using NBS to give final compound 5 and 7, respectively. (4,4-bis(2-ethylhexyl)-4H-cyclopenta[2,1-b:3,4-b′]dithiophene-2,6-diyl)bis(trimethylstannane) (CPDT, 8) co-monomer was synthesized from the previously reported synthetic procedures [21,36]. All the synthesized monomers 3, 5, and 7 were identified by ^1^H nuclear magnetic resonance spectroscopy (NMR), whose spectra were recorded in Supporting Information (Figures S1–S5).

Three BBO-based novel conjugated polymers were synthesized by Still polycondensation of monomer 8 (CPDT) with monomer 3, 5, and 7, and the resulting polymers were named PBC1, PBC2, and PBC3, respectively. PBC1 is the simplest structure composed of CPDT and BBO building blocks, whereas PBC2 and PBC3 have π-extended polymeric backbone structures with additional thiophene π-spacers. To investigate the effect of the alkyl side chain on the π-spacer, both PBC2 with a non-alkylated thiophene π-spacer and PBC3 with an octylthiophene π-spacer were synthesized. The detailed synthetic routes of PBC1, PBC2, and PBC3 were shown in Scheme 1.

The molecular weights of the synthesized polymers were evaluated using gel permeation chromatography (GPC). The number average molecular weights of PBC1, PBC2, and PBC3 were measured to be 13,400 (*Đ* = 2.28), 13,500 (*Đ* = 2.29), and 8400 (*Đ* = 1.53), respectively, and their GPC spectra were recorded in Figures S9–S11. PBC3 showed slightly lower molecular weight than the other polymers, which is expected to be from the steric hindrance between alkylated monomer 7 and CPDT.

### 3.2. Optical and Electrochemical Properties

**Figure 1:**
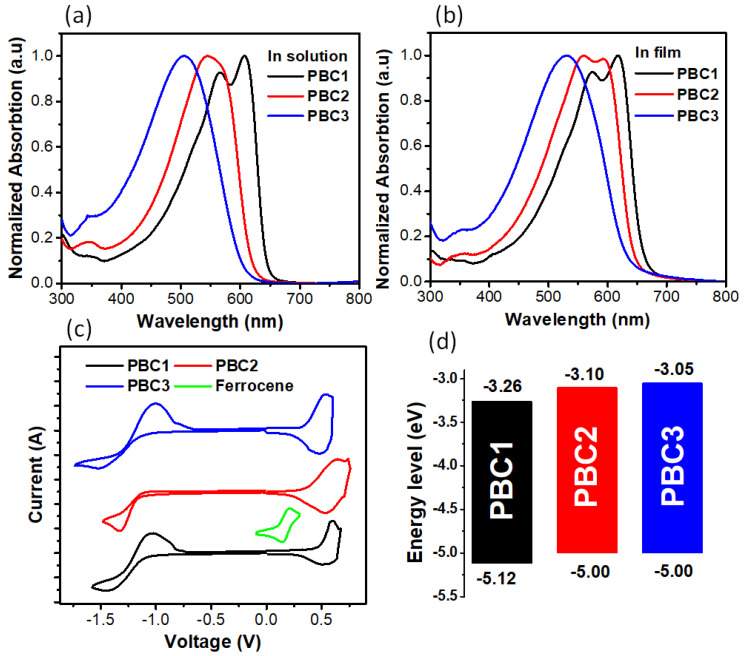
UV-Vis spectroscopy of the synthesized PBC polymers (**a**) in solution and (**b**) in film. (**c**) Cyclic voltamogram of the PBC polymers, and (**d**) the estimated HOMO and LUMO energy levels.

The UV-Vis absorption spectra of PBC1, PBC2, and PBC3 were measured in dilute solution and film states. As shown in Figure 1a,b and Table 1, the maximum absorption peaks of PBC1, PBC2, and PBC3 appeared at 564 (606), 544, and 504 nm, respectively, in the solution, and 575 (617), 560 (592) and 530 nm, respectively, in film states. The absorption peaks of PBC1 were the most red-shifted in both solution and film states. This indicates that intramolecular charge transport interaction between CPDT (donor unit) and BBO (acceptor unit) was the most effective without π-spacers. In the comparison of PBC2 and PBC3, the absorption peak of PBC3 containing an alkylated π-spacer was more blue-shifted than that of PBC2 having a non-alkylated π-spacer. Moreover, the PBC3 polymer did not show shoulder absorption in the solution or films. This indicates that the alkyl side chains on the thiophene π-spacer induced a more twisted backbone structure of PBC3 and interrupted molecular ordering of PBC3 in the film states. The absorption peaks of the polymers in film states were more red-shifted than those in the solution, and the degree of red-shift in the absorption maximum peak of PBC1, PBC2, and PBC3 (solution → film) was 11, 47, and 23 nm, respectively. This indicates that all the polymers make better intermolecular interaction in the film states, and notably, PBC2 having a non-alkylated thiophene π-spacer makes the strongest intermolecular interaction and ordering in the film states.

The energy levels of the polymers were measured from cyclic voltammetry (CV) and their cyclic voltammogram were shown in Figure 1c. The energy level of the ferrocene/ferrocenium (Fc/Fc^+^) reference electrode was assumed as −4.8 eV to vacuum, and its half-wave potential (E_1/2_) was measured to be 0.15 V. The highest occupied molecular orbital (HOMO) energy level was estimated from the oxidation onset potential (*E*_ox_) [37,38]. The *E*_ox_ of PBC1, PBC2, and PBC3 were 0.493, 0.367, and 0.369 V, respectively, which corresponds to the HOMO energy level (*E*_HOMO_) of −5.12, −5.00, and −5.00 eV, respectively. The lowest unoccupied molecular orbital (LUMO) energy level of PBC1, PBC2, and PBC3 was estimated from the reduction onset potential (*E*_re_), and their values were −1.108, −1.160, and −1.139 V, respectively, which correspond to LUMO energy level (E_LUMO_^CV^) of −3.52, −3.47 and −3.49 eV, respectively. The LUMO energy level (*E*_LUMO_^Opt^) was also calculated from the *E*_HOMO_ and optical bandgap of the polymers, and the *E*_LUMO_^Opt^ of PBC1, PBC2, and PBC3 was −3.26, −3.10, and −3.05 eV, respectively. The values of *E*_LUMO_^CV^ and *E*_LUMO_^Opt^ are quite different due to the different measuring methods, but their trends are similar to each other. In a comparison of PBC2 and PBC3, their conjugated backbone structures are identical, thus there is no significant difference in the energy levels (−5.0 eV). On the other hand, PBC1 has a deeper HOMO energy level (−5.12 eV) than those PBC2 and PBC3. This indicates that the introduction of an electron-donating thiophene π-spacer to the polymer chain effectively upshifts the HOMO energy level of the polymer, which is beneficial for hole transport in the devices. The energy level diagram and CV data were summarized in Figure 1d and Table 1, respectively.

### 3.3. OTFT Properties and Analysis

**Figure 2:**
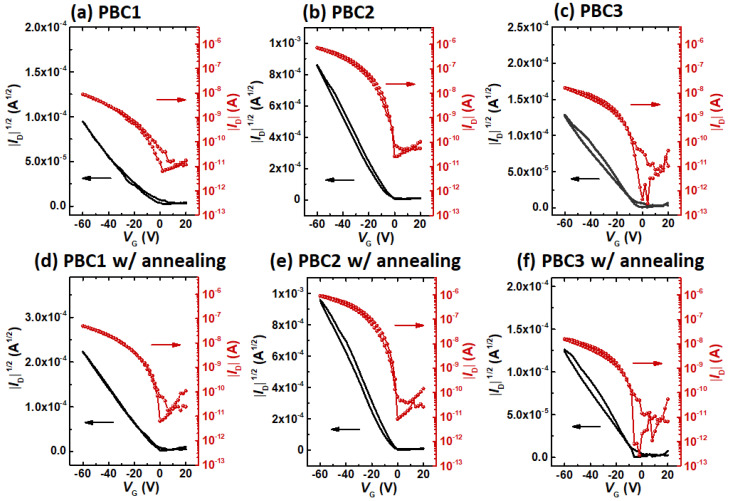
Charge transfer curves of (**a**,**d**) PBC1, (**b**,**e**) PBC2, and (**c**,**f**) PBC3 devices in as-casted films (**a**–**c**) and after thermal annealing ((**d**): 200 °C, (**e**): 100 °C, (**f**): 100 °C).

The top contact/bottom gate OTFTs were fabricated to study the hole-transporting characteristics of the synthesized polymers. The transfer and output characteristics of the OTFTs are shown in Figure 2 and Figure S12, respectively. All the polymers showed typical *p*-type charge transporting characteristics, and the maximum hole mobility of PBC1, PBC2, and PBC3 in the as-cast films was 1.3 × 10^−4^, 1.5 × 10^−2^ and 4.7 × 10^−4^ cm^2^/Vs, respectively. Notably, PBC2 showed 100 times higher hole mobility than those PBC1 and PBC3. Since hole mobility can be changed depending on the surface roughness, morphology, and temperature [39,40,41], the charge-transporting properties of the polymers were further optimized by the post-thermal annealing process from the as-cast films. The hole mobility of PBC1 was significantly increased to 9.2 × 10^−4^ cm^2^/Vs via thermal annealing at 200 °C, which indicates that the molecular ordering was highly improved after thermal annealing. On the other hand, the hole mobility of PBC3 was not increased at all under thermal treatment, which implies weak crystallinity of PBC3. In the case of PBC2, the hole mobility was increased up to 2.2 × 10^−2^ cm^2^/Vs and the on/off ratio reached 10^5^ via thermal annealing at 100 °C. The hole-transporting properties of PBC2 were superior to those of the other polymers, which strongly implies that PBC2 has the most preferred molecular ordering for charge transport in OTFTs. After annealing at 150 °C, lower mobility was observed compared to the as-cast condition. As seen in Figure S13, there was a significant change in surface morphology. At the as-cast film and 100 °C, a granular structure was well-formed, but it was greatly reduced and numerous holes began to appear due to the decomposition at 150 °C. OTFT properties of the polymers are summarized in Table 2.

**Figure 3:**
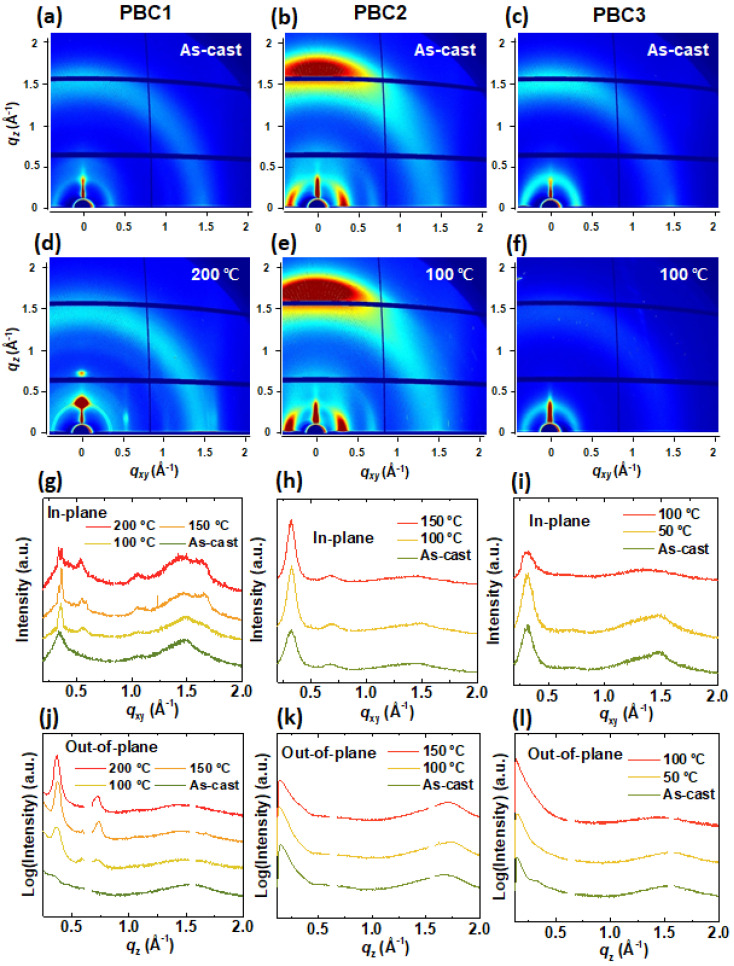
Two-dimensional grazing incidence X-ray diffraction patterns of as-casted (**a**) PBC1, (**b**) PBC2 and (**c**) PBC3 films, and thermally-annealed (**d**) PBC1, (**e**) PBC2 and (**f**) PBC3 films. The corresponding line-cut profiles of (**g**,**j**) PBC1, (**h**,**k**) PBC2, and (**i**,**l**) PBC3 films along the in-plane (*q*_xy_) axes (**g**–**i**) and out-of-plane (*q*_z_) and (**j**–**l**).

To understand the difference in the charge transporting characteristics of the synthesized polymers, 2D grazing incidence wide-angle X-ray diffraction (2D-GIXD) of the polymer films was investigated before and after thermal annealing (Figure 3 and Figure S14–S16) [42]. Figure 3 shows 2D-GIXD images and the corresponding line-cut spectra along the in-plane (*q*_xy_) and out-of-plane (*q*_z_) directions. As shown in Figure 3a–c, it is clearly observed that only PBC2 showed strong molecular ordering and orientation in the film state, whereas PBC1 and PBC3 polymers showed featureless ordering behaviors in the film state.

In detail, as shown in Figure 3b,e,h,k, the lamellar ordering reflection (100, 200) peaks of PBC2 clearly appeared at 0.33 and 0.67 Å^−1^ along the *q*_xy_ axis, respectively, which corresponds to d-spacing of 19 Å in the lamellar ordering. The π-π stacking (010) peak was also observed at 1.69 Å^−1^ along the *q*_z_ axis, indicating π-π stacking d-spacing of 3.7 Å. After thermal annealing at 100 °C, the intensity of lamellar ordering (100, 200) peaks are increased, and (010) peak was shifted to 1.73 Å^−1^ (π-π stacking d-spacing: 3.6 Å). This indicates that the lamellar structure of PBC2 is more developed, and π-π stacking interaction among polymer backbones becomes stronger after thermal annealing. This high crystallinity and strong molecular ordering could result in the superior hole mobility of PBC2.

As shown in Figure 3a,d,g,j, the molecular ordering of PBC1 is relatively weak, but it showed the mixed molecular orientation in both face-on and edge-on modes. (100) peaks appeared at 0.35 Å^−1^ and 0.34 Å^−1^ along the *q*_xy_ and *q*_z_ axis, respectively, and the lamellar d-spacing was 18 Å. (010) peaks were observed at approximately 1.5 Å^−1^, indicating π-π stacking d-spacing of 4.2 Å. PBC1 film showed relatively large π-π stacking distance, and weak intermolecular ordering compared to those of PBC2 film. However, similar to PBC2, the lamellar and π-π stacking ordering of PBC1 was effectively enhanced by thermal annealing. As shown in Figure 3g,j, (100) peaks are shifted to ~0.37 Å^−1^ along the *q*_xy_ and *q*_z_ axis after annealing at 200 °C, and the new ordering patterns appeared at 0.54 and 1.66 Å^−1^ along the *q*_xy_ axis. The thermal annealing on PBC1 films effectively improved molecular ordering and orientation, which is in good agreement with the enhanced hole mobility of PBC1 via thermal treatment.

As shown in Figure 3c,f,i,l, PBC3 showed quite weak (100) and (010) peaks at 0.32 Å^−1^ and 1.48 Å^−1^, respectively, which correspond to d-spacing of 19.6 Å (lamellar) and 4.2 Å (π-π stacking), respectively. In addition, the thermal annealing process rather reduced the molecular ordering of PBC3 film. This means that PBC3 has the weakest intermolecular interactions and low crystallinity, resulting in the lowest hole mobilities among the three polymers.

**Figure 4:**
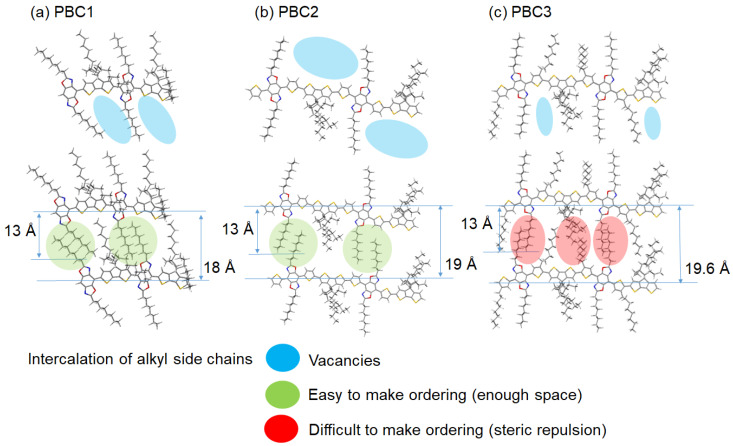
Optimized two repeating unit structures of (**a**) PBC1, (**b**) PBC2, and (**c**) PBC3 simulated by density-functional theory (DFT) (B3LYP/6-31G), and the expected lamellar ordering based on the DFT simulation and d-spacing values.

To understand the relationship between the polymer structure and hole-transporting properties, two repeating units of PBC1, PBC2, and PBC3 were simulated by density-functional theory (DFT) (B3LYP/6-31G). As shown in Figure 4, we found that intercalation between the alkyl side chains is present in all polymer films because the length of alkyl side chains from the polymer backbone is approximately 13 Å, and the lamellar d-spacing is 18~19 Å. In the case of PBC1, there are vacancies between alkyl side chains on the polymer backbone and thus the intercalation of alkyl side chains happens to increase the molecular ordering of PBC1. The addition of non-alkylated thiophene π-spacers to the polymer backbones increases these vacancies between CPDT and BBO repeating units, allowing for more efficient intercalation of alkyl side chains and improving intermolecular ordering and crystallinity of PBC2. However, the addition of alkylated thiophene π-spacers to the polymer backbones rather reduces these vacancy sites between CPDT and BBO repeating units, preventing efficient intercalation of alkyl side chains and giving PBC3 low crystallinity. Thus, the molecular ordering and hole mobility was increased in the order of PBC3 < PBC1 < PBC2. The introduction of a non-alkylated π-spacer unit between CPDT and BBO building blocks was crucial to improving the hole mobility of the polymers. The annealing effect was also related to the ordering of alky side chains. In the case of PBC1 and PBC2, thermal annealing makes more optimal intercalation of alkyl side chains, which increases the molecular ordering and hole mobility in the film states. However, in the case of PBC3, thermal energy during the annealing process rather increases the steric repulsion between the alkyl side chains due to the inefficient intercalation of alkyl side chains on the polymer backbones. Thus, only the hole mobilities of PBC1 and PBC2 devices were enhanced via thermal annealing.

**Figure 5:**
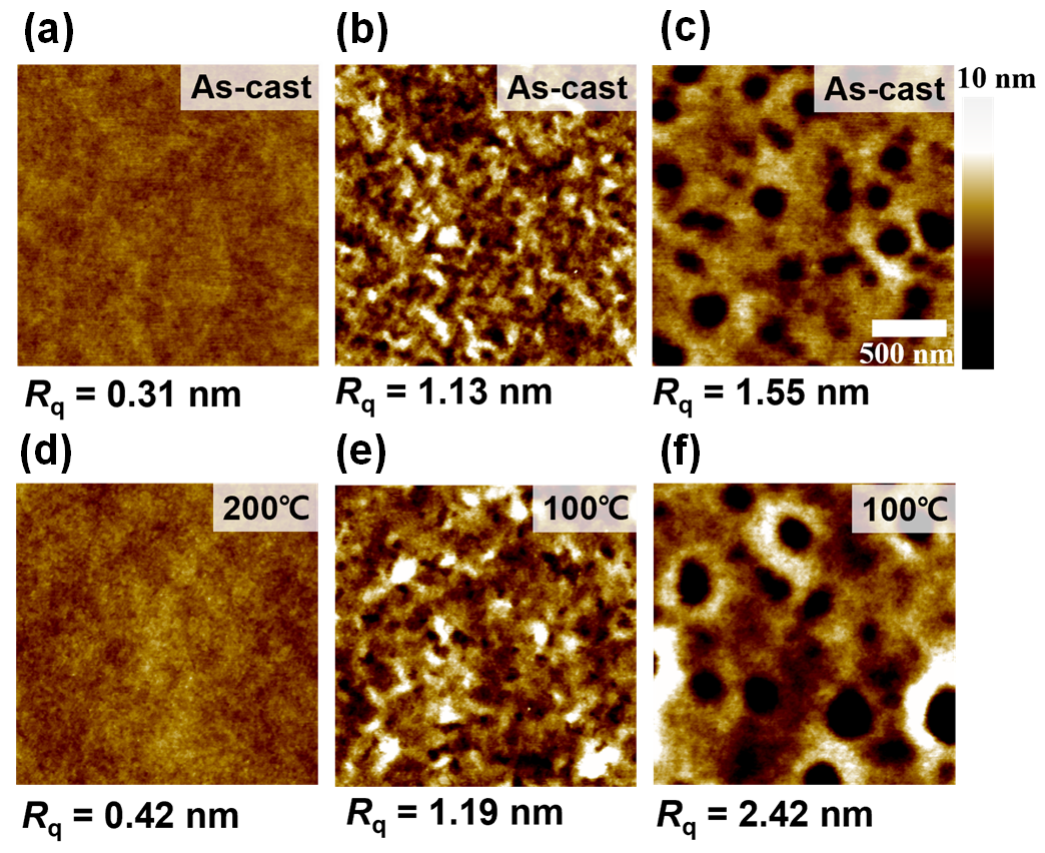
AFM images of (**a**,**d**) PBC1, (**b**,**e**) PBC2, and (**c**,**f**) PBC3 in as-casted films (**a**–**c**) and after thermal annealing (**d**–**f**).

Topographic images of the as-cast and thermally annealed PBC1, PBC2, and PBC3 films were measured by atomic force microscopy (AFM) (Figure 5). At as-cast films, PBC1, PBC2, and PBC3 films showed the highest root-mean-square surface roughness (*R*_q_) of 0.31, 1.13, and 1.55 nm, respectively. PBC1 showed highly uniform surface morphology, whereas PBC2 showed highly ordered nanomorphology. In other words, PBC2 films seem to have sizable crystallinity, whereas PBC1 films have weak molecular ordering and crystallinity [43,44]. In the case of PBC3, it showed relatively poor surface images. After thermal annealing, the *R*_q_ values of PBC1, PBC2, and PBC3 were slightly increased to 0.42, 1.19, and 2.42 nm, respectively, but there was no significant difference.

## 4. Conclusions

We synthesized three novel BBO-based conjugated polymers to study hole-transporting characteristics in OTFTs. PBC1 is the simplest structure composed of CPDT and BBO building blocks, whereas PBC2 and PBC3 have π-extended polymeric backbone structures with additional thiophene π-spacers. To investigate the effect of the alkyl side chain on the π-spacer, both PBC2 with a non-alkylated thiophene π-spacer and PBC3 with an octylthiophene π-spacer were synthesized. The top contact/bottom gate OTFTs were fabricated to study hole transporting characteristics of PBC polymers, and PBC2 devices showed the highest hole mobility of 2.2 × 10^−2^ cm^2^ V^−1^ s^−1^, which was 100 times higher than the PBC1 and PBC3 devices. PBC1 showed weak molecular ordering in the film states, thus the introduction of a π-spacer unit between CPDT and BBO was important to control the molecular ordering of the polymers. PBC2 showed the strongest molecular ordering and crystallinity, whereas PBC3 almost lose its crystallinity. From the 2D-GIXD and DFT calculation, we found that the non-alkylated thiophene π-spacer provides empty spaces between CPDT and BBO repeating units. Thus, the alkyl side chains on the conjugated backbone can be easily intercalated with each other, which increased the intermolecular ordering and hole mobility. On the other hand, the alkylated thiophene π-spacers fill the empty space between CPDT and BBO repeating units, which prevents intercalation of alkyl side chains on the conjugated backbones and decreases the intermolecular ordering and hole mobility. BBO conjugated building block has great potential to utilize in OTFTs and the selection of conjugated π-spacer in BBO-based polymers is important to improve OTFT performances.

## Figures and Tables

**Table 1:** Optical and electrochemical properties of PBC1, PBC2, and PBC3.

Polymer	λ_max_ (nm) ^a^		*E*_g_^opt^ (eV) ^b^	*E*_ox_ (V)/*E*_HOMO_^CV^ (eV) ^c^	*E*_re_ (V)/*E*_LUMO_^CV^ (eV) ^d^	*E*_LUMO_^Opt^ (eV) ^e^
	Solution	Film				
PBC1	564, 606	575, 617	1.86	0.47/−5.12	−1.12/−3.53	−3.26
PBC2	544	560, 592	1.90	0.35/−5.00	−1.18/−3.47	−3.10
PBC3	504	530	1.95	0.35/−5.00	−1.17/3.48	−3.05

^a^ Maximum absorption wavelength; ^b^ optical bandgap; ^c^ HOMO energy level from CV; ^d^ LUMO energy level from CV; ^e^ LUMO energy level from *E*_HOMO_^CV^ and *E*_g_^opt^. *E*_HOMO_^CV^ = −4.8 − (E_ox_ − E_1/2_) eV; *E*_LUMO_^CV^ = −4.8 − (E_re_ − E_1/2_) eV.

**Table 2:** Summary of OTFT performances.

Polymer	Annealing Temperature (°C)	Field-Effect Mobility (*μ)* (cm^2^ V^−1^·s^−1^)	On/Off Ratio	Threshold Voltage (V)
PBC1	As-cast	0.00013	10^3^	−3.9 ± 5.4
	100	0.00045	10^3^	−2.8 ± 1.2
	150	0.00080	10^3^–10^4^	−3.4 ± 0.5
	200	0.00092	10^3^–10^4^	−2.9 ± 1.5
PBC2	As-cast	0.015	10^4^	−2.9 ± 0.3
	100	0.022	10^5^	−3.1 ± 1.1
	150	0.011	10^5^	−3.4 ± 0.7
PBC3	As-cast	0.00047	10^3^	−3.5 ± 1.1
	50	0.00050	10^3^	−4.3 ± 0.8
	100	0.00034	10^3^	−5.8 ± 2.9

## Data Availability

Data are available upon request.

## Supplementary Materials

The following supporting information can be downloaded at: https://www.mdpi.com/article/10.3390/polym15051156/s1, Figure S1: ^1^H NMR of compound 3 (BBO); Figure S2: ^1^H NMR of compound 4; Figure S3: ^1^H NMR of compound 5; Figure S4: ^1^H NMR of compound 6; Figure S5: ^1^H NMR of compound 7; Figure S6: ^1^H NMR of PBC1; Figure S7: ^1^H NMR of PBC2; Figure S8: ^1^H NMR of PBC3; Figure S9: GPC spectrum of PBC1; Figure S10: GPC spectrum of PBC2; Figure S11: GPC spectrum of PBC3; Figure S12: Output characteristics of (a,d) PBC1, (b,e) PBC2 and (c,f) PBC3 devices in as-casted films (a,b,c) and after thermal annealing (d: 200 °C, e: 100 °C, f: 100 °C); Figure S13: Atomic force microscopy (AFM) topography (2 μm × 2 μm) of the PBC2 thin films at the as-cast, 100 °C, and 150 °C annealing conditions; Figure S14: Two-dimensional grazing incidence X-ray diffraction (2D-GIXD) patterns of PBC1 depending on the thermal annealing temperature; Figure S15: 2D-GIXD patterns of PBC2 depending on the thermal annealing temperature; Figure S16: 2D-GIXD patterns of PBC3 depending on the thermal annealing temperature.
