# Supplementary material for: Development of Benzobisoxazole-Based Novel Conjugated Polymers for Organic Thin-Film Transistors
Source: Polymers (Basel). 2023 Feb 24;15(5):1156. doi: 10.3390/polym15051156 (PMC10007153; doi:10.3390/polym15051156)
Supplement: Supplementary file 1 [file polymers-15-01156-s001.zip › polymers-2223070-supplementary.pdf]

polymers

MDPI

Supporting Information

Development of Benzobisoxazole-Based Novel Conjugated Polymers for Organic Thin-Film Transistors

WonJo Jeong [1,†], Kyumin Lee [2,†], Jaeyoung Jang [2,*] and In Hwan Jung [1,*]

1 Human-Tech Convergence Program, Department of Organic and Nano Engineering, Hanyang University, 222 Wangsimni-ro, Seongdong-gu, Seoul 04763, Republic of Korea
2 Department of Energy Engineering, Hanyang University, Seoul 04763, Republic of Korea
* Correspondence: jyjang15@hanyang.ac.kr (J.J.); inhjung@hanyang.ac.kr (I.H.J.)
† These authors contributed equally to this work.

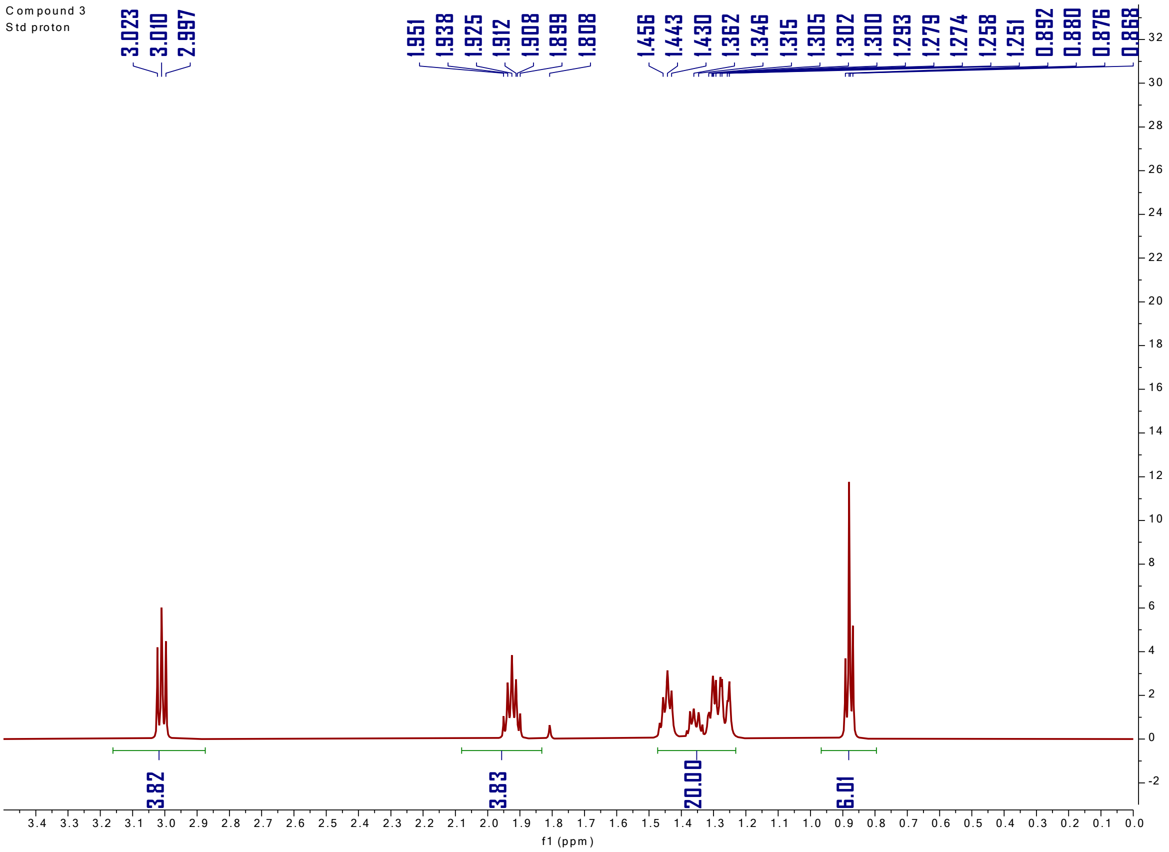

Figure S1. ^{1}H NMR of compound 3 (BBO).

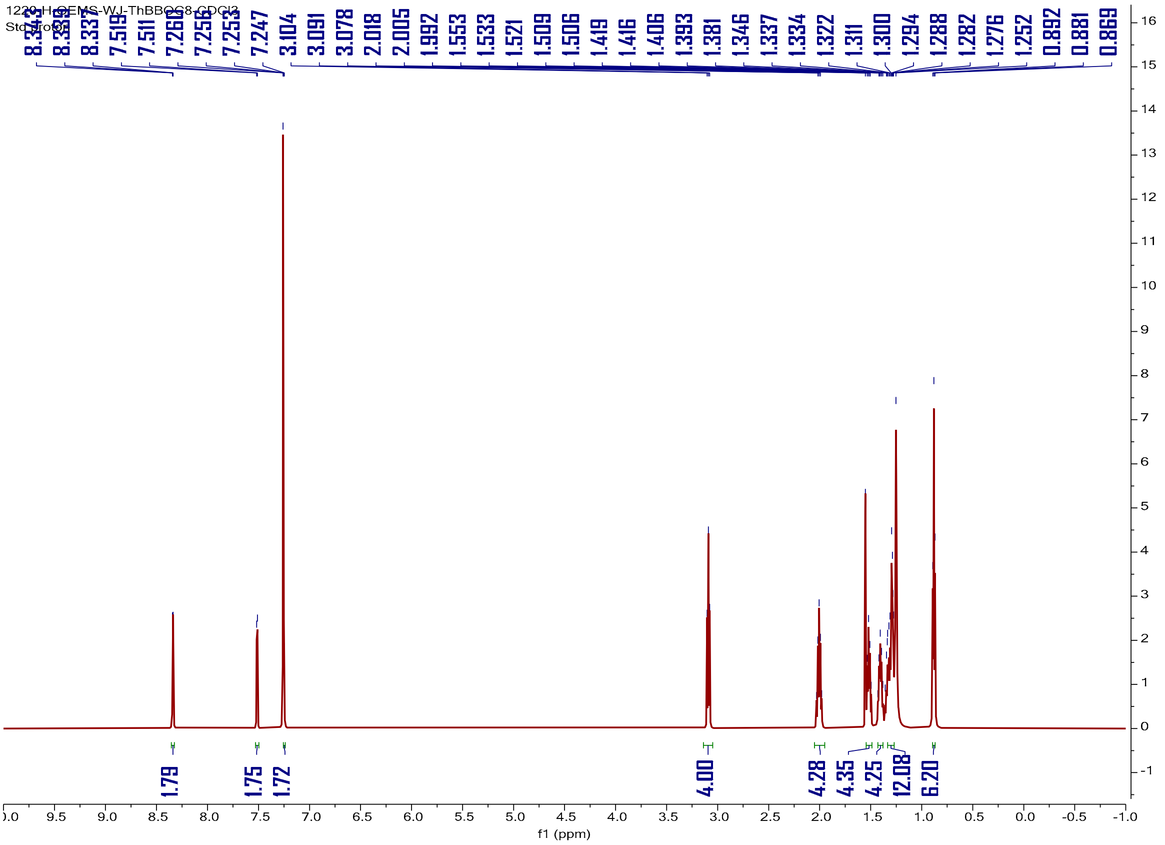

Figure S2. ^{1}H NMR of compound 4.

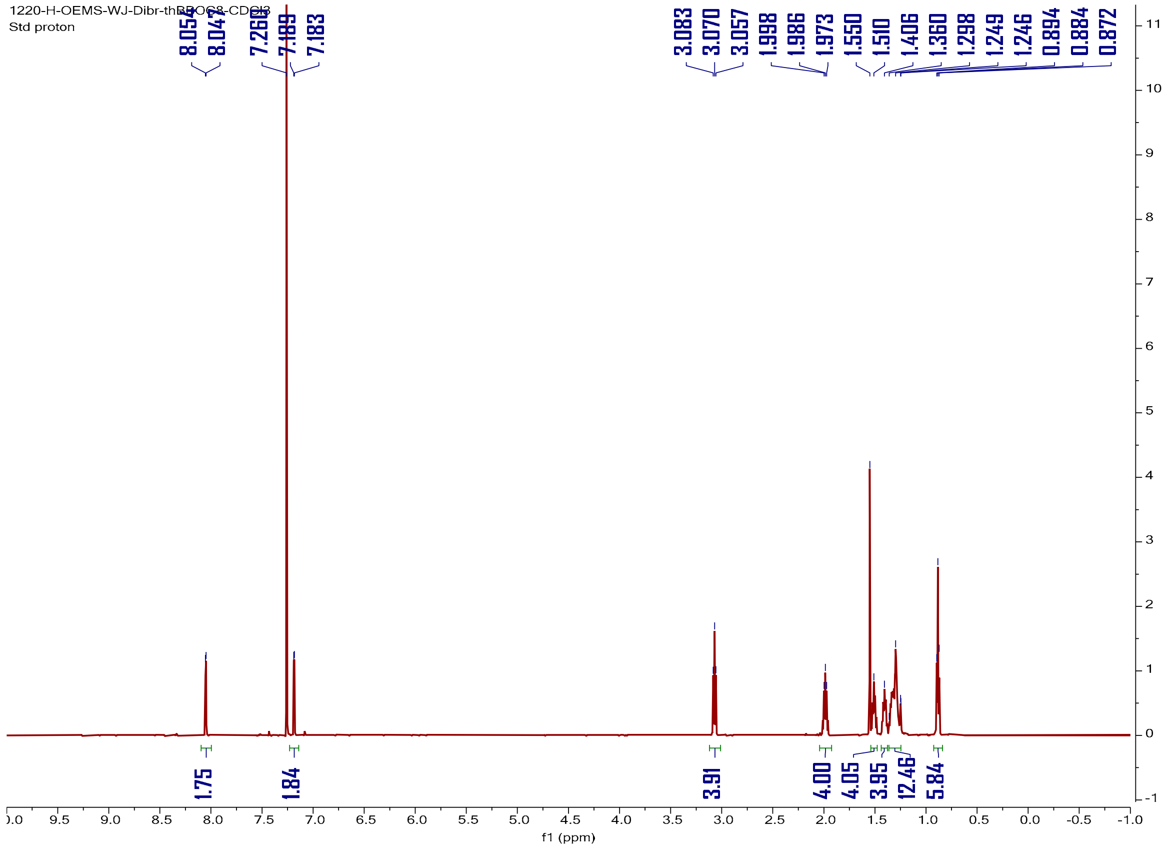

Figure S3. ^{1}H NMR of compound 5.

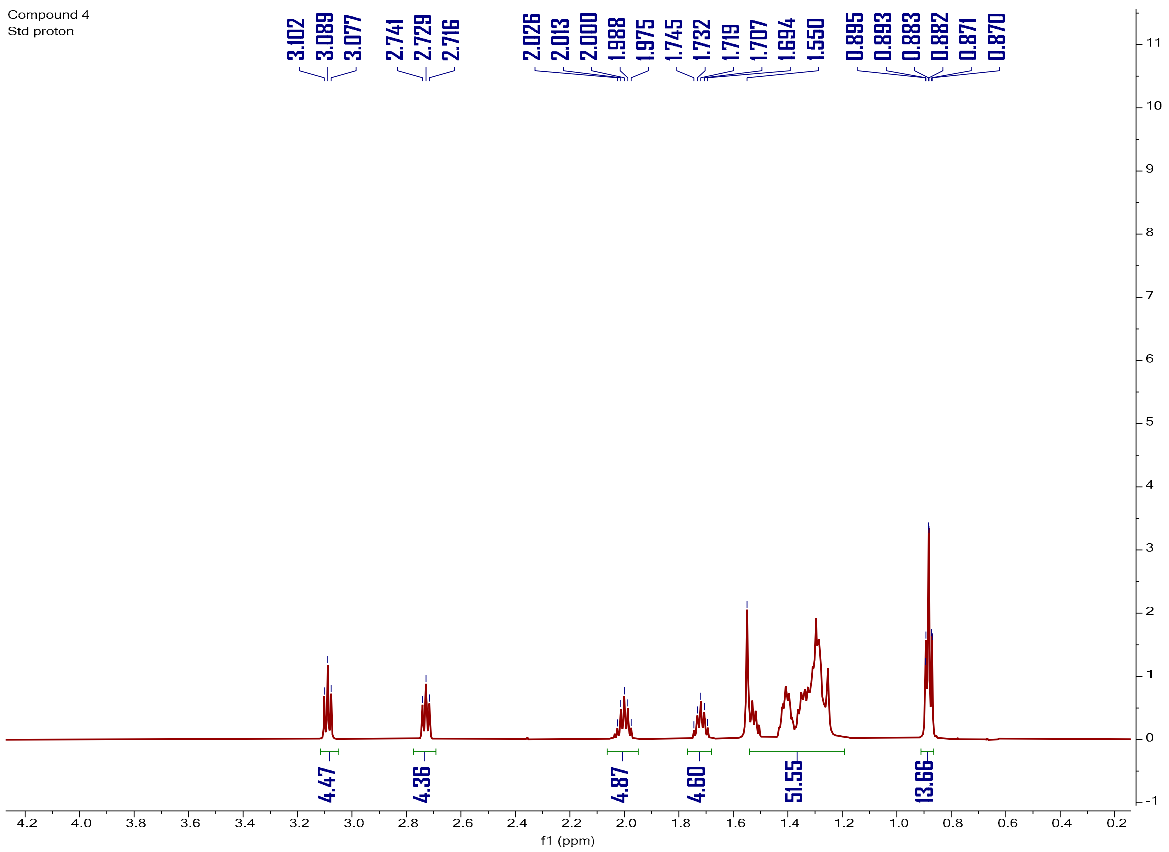

Figure S4. ^{1}H NMR of compound 6.

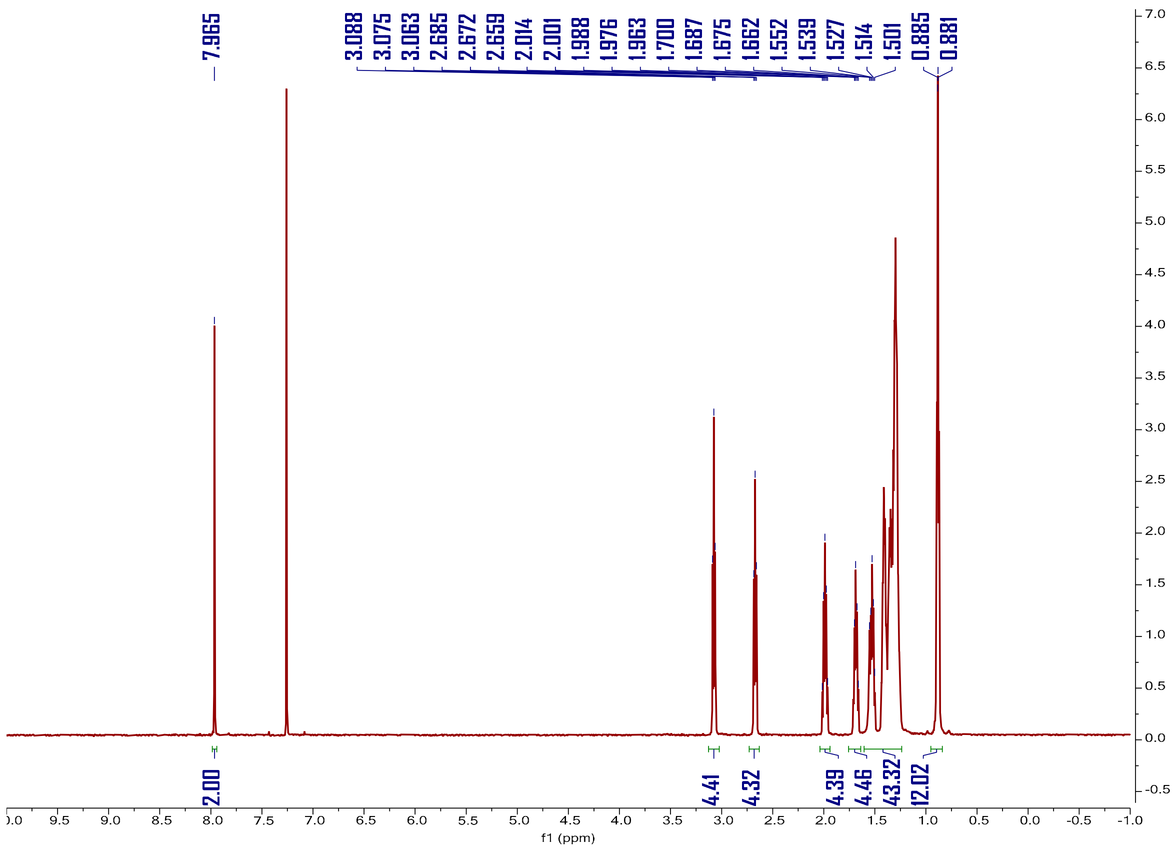

Figure S5. ^{1}H NMR of compound 7.

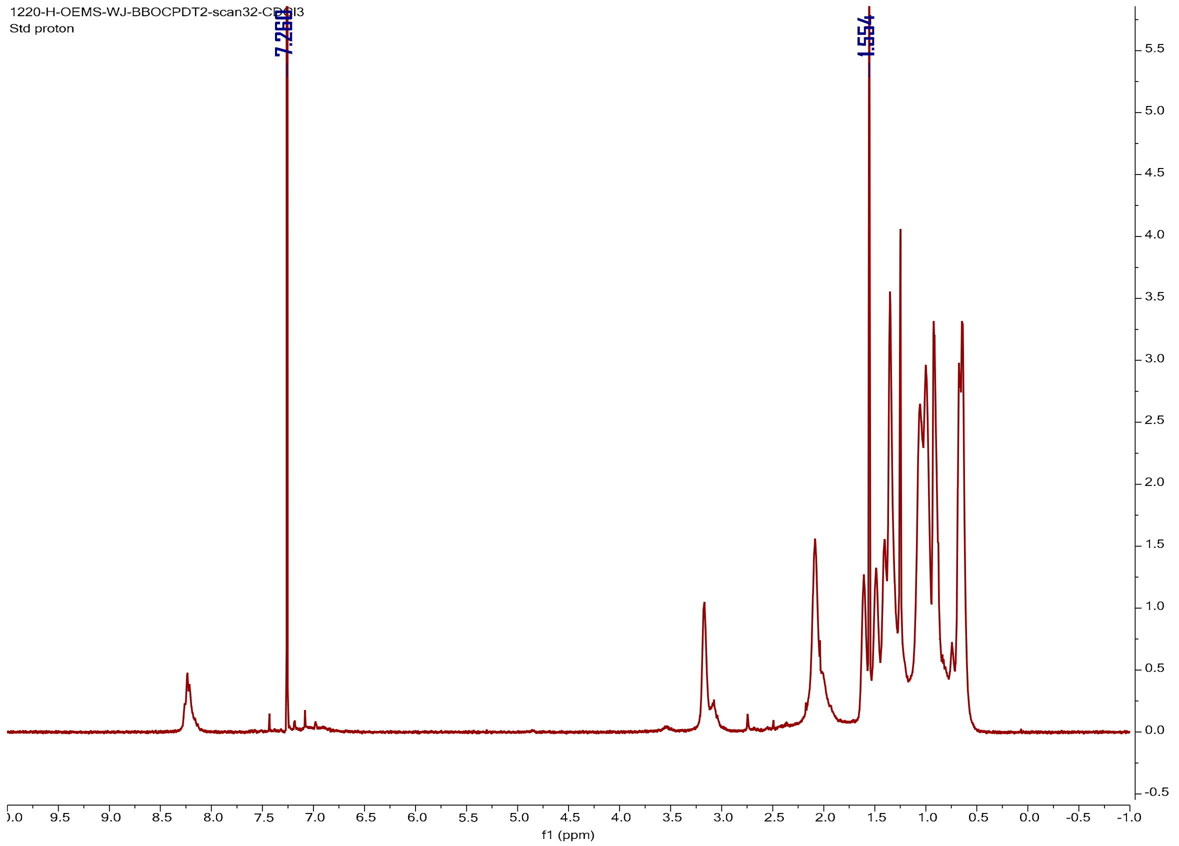

Figure S6. ^{1}H NMR of PBC1.

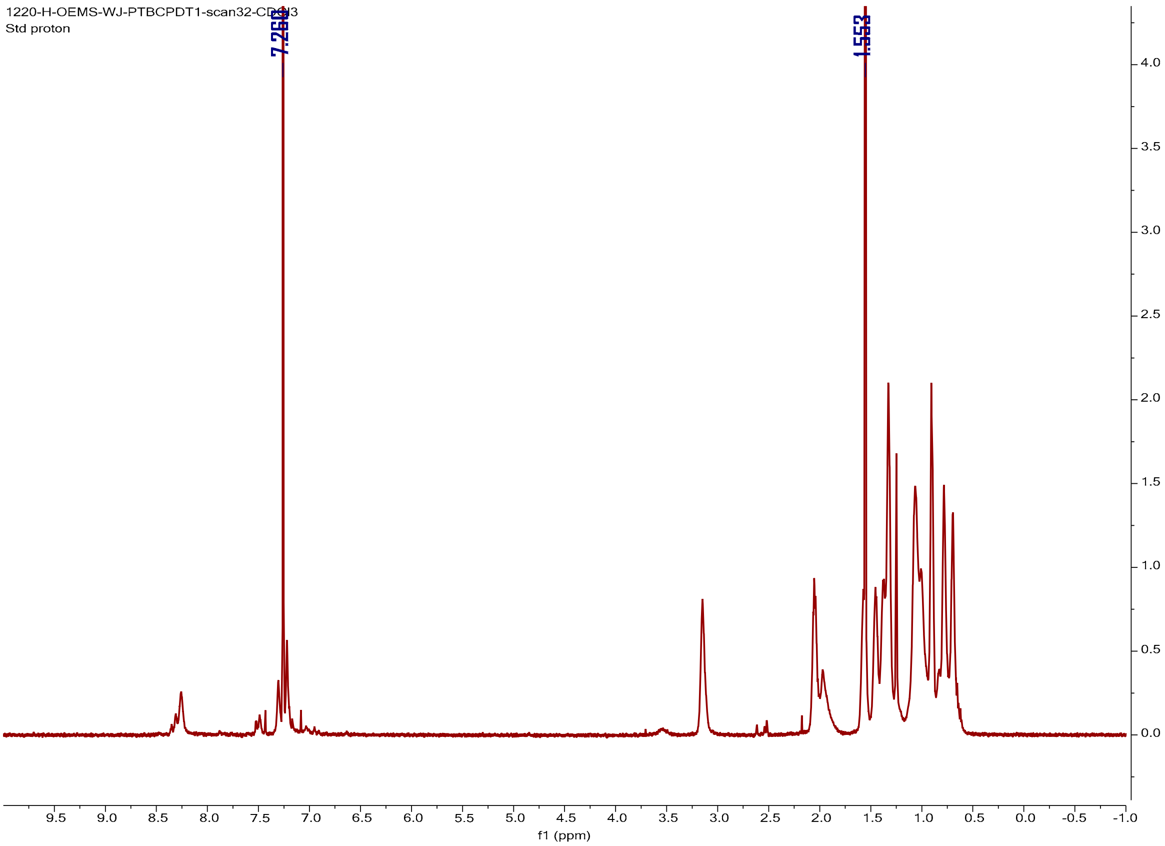

Figure S7. ^{1}H NMR of PBC2.

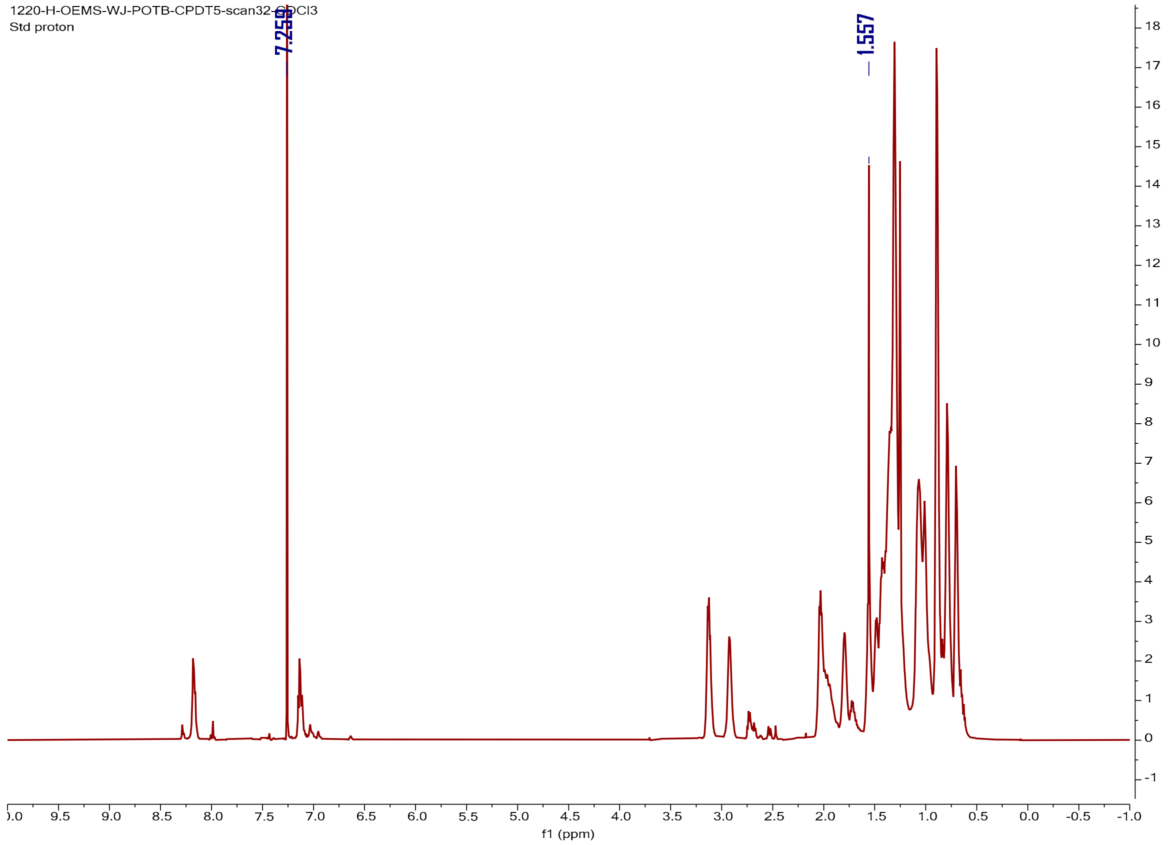

Figure S8. ^{1}H NMR of PBC3.

Molecular Weight Averages

Peak #	RT (min)	Mp (g/mol)	Mn (g/mol)	Mw (g/mol)	Mz (g/mol)	Mz+1 (g/mol)	PD
1	6.657	20470	13357	30427	58241	88174	2.277982

Chromatogram

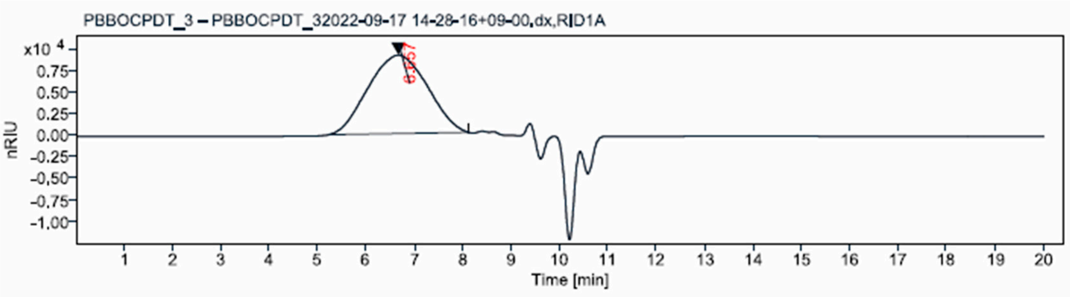

Figure S9. GPC spectrum of PBC1.

Molecular Weight Averages

Peak #	RT (min)	Mp (g/mol)	Mn (g/mol)	Mw (g/mol)	Mz (g/mol)	Mz+1 (g/mol)	PD
1	6.656	20730	13511	30958	60091	92441	2.291318

Chromatogram

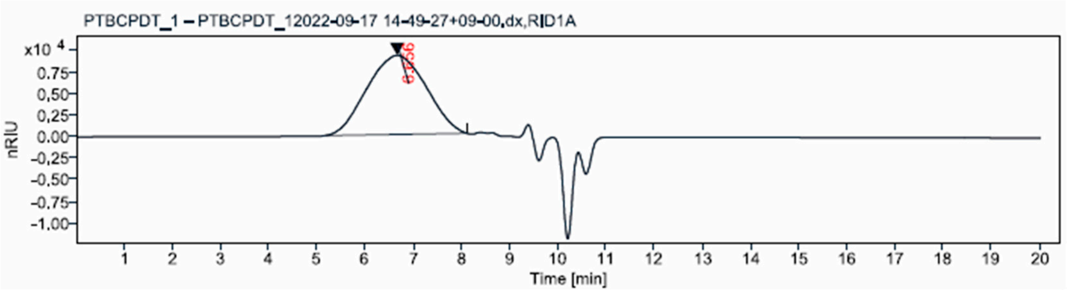

Figure S10. GPC spectrum of PBC2.

Molecular Weight Averages

Peak #	RT (min)	Mp (g/mol)	Mn (g/mol)	Mw (g/mol)	Mz (g/mol)	Mz+1 (g/mol)	PD
1	7.039	10695	8352	12738	18463	24652	1.525144

Chromatogram

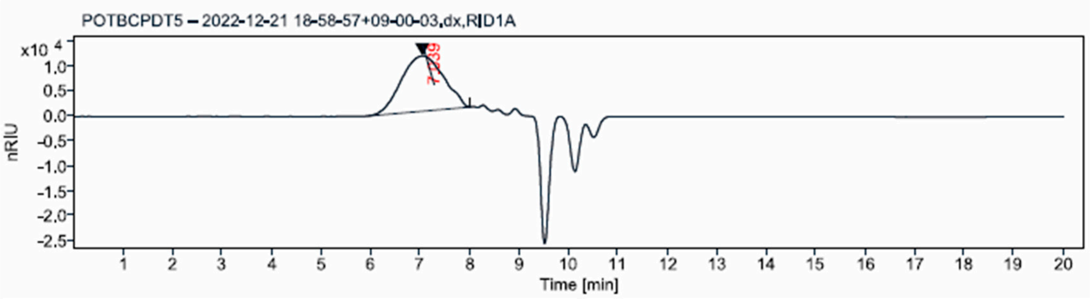

Figure S11. GPC spectrum of PBC3.

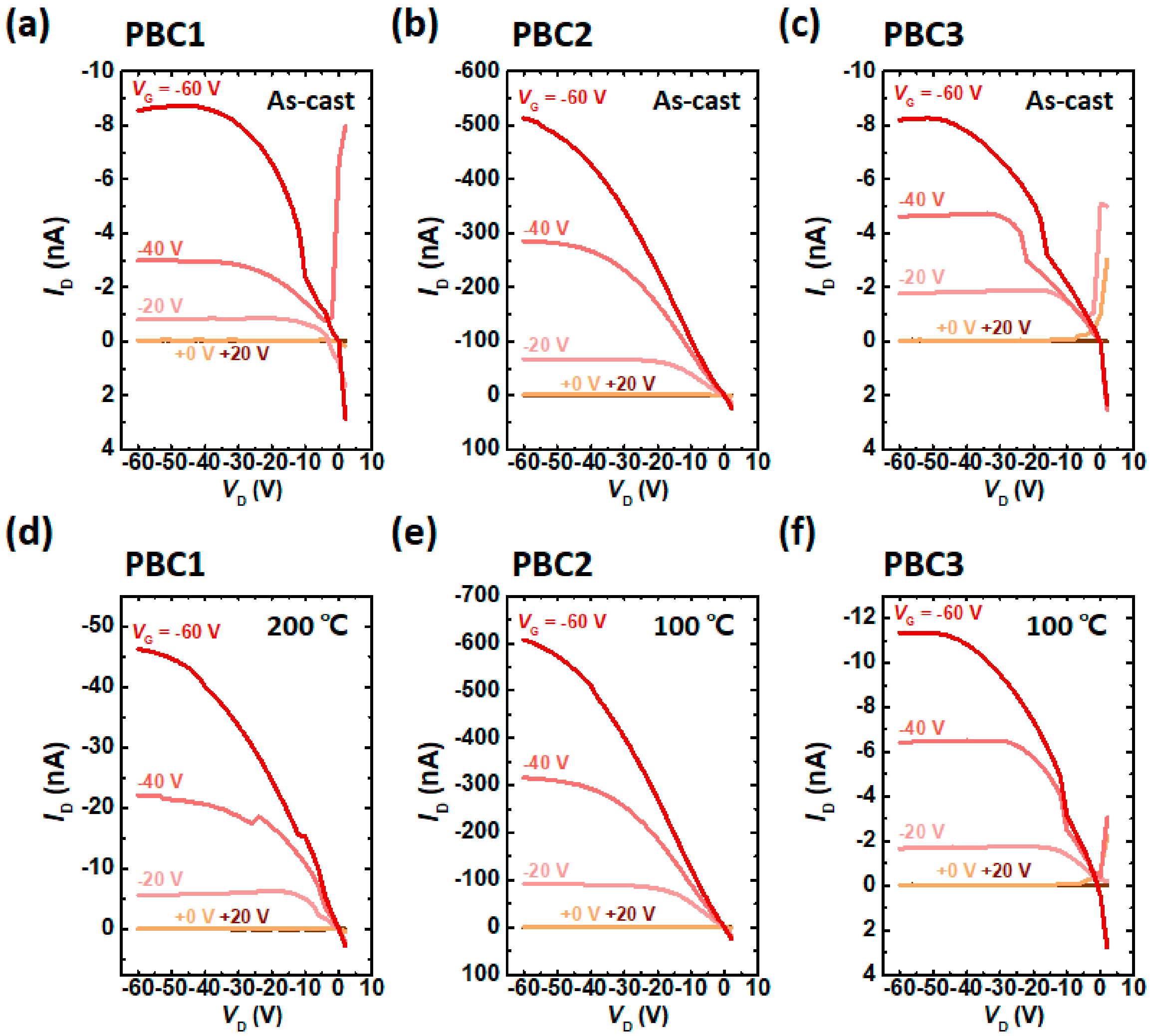

Figure S12. Output characteristics of (a, d) PBC1, (b, e) PBC2, and (c, f) PBC3 devices in as-casted films (a, b, c) and after thermal annealing at the optimum temperature (d: 200 ℃, e: 100 ℃, f: 100 ℃).

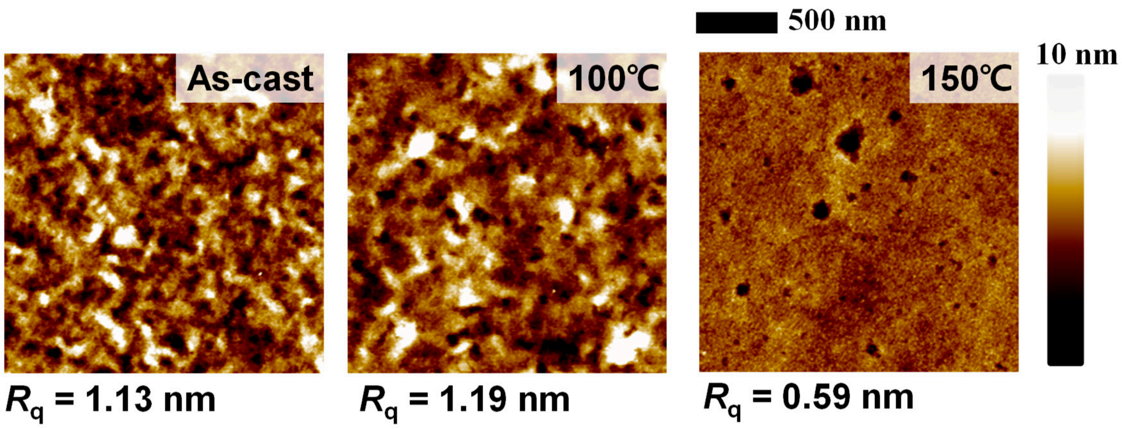

Figure S13. Atomic force microscopy (AFM) topography (2 μm × 2 μm) of the PBC2 thin films at the as-cast, 100°C, and 150°C annealing conditions.

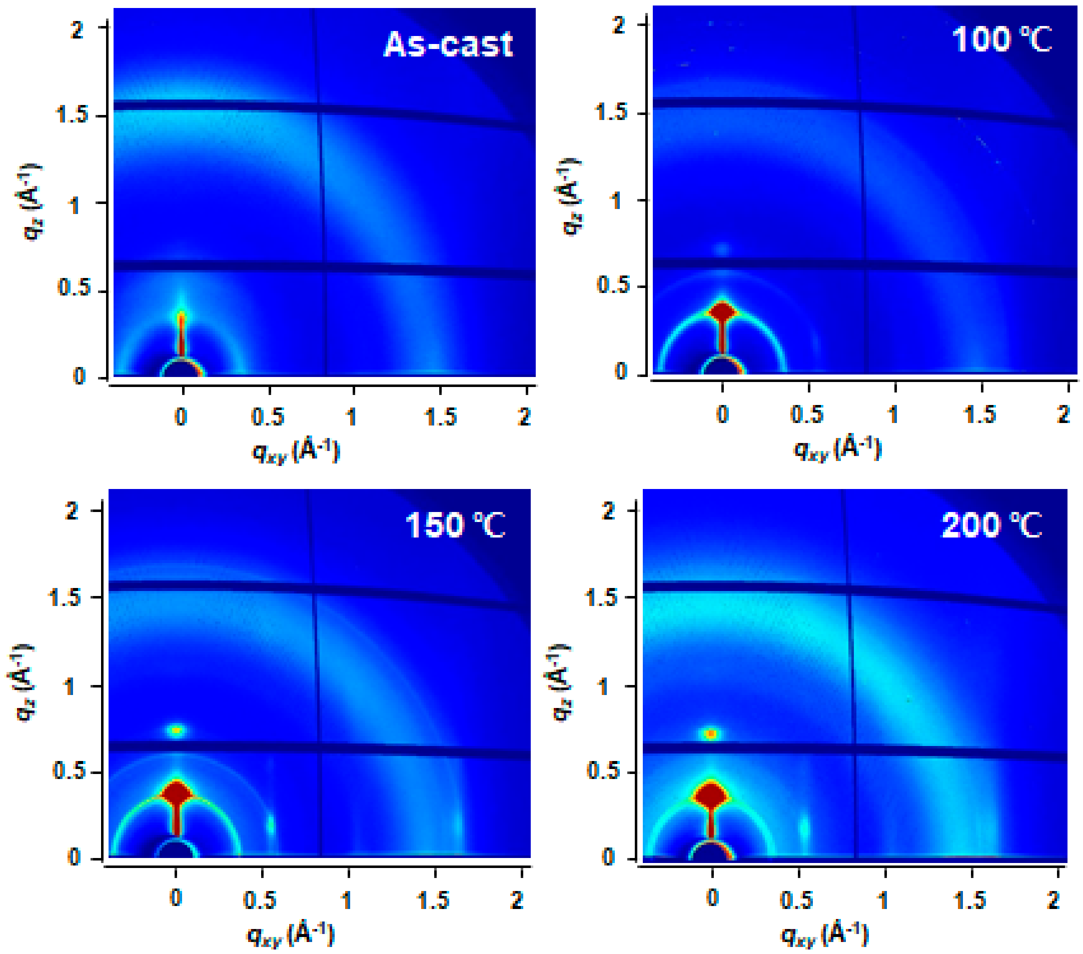

Figure S14. Two-dimensional grazing incidence X-ray diffraction (2D-GIXD) patterns of PBC1 depending on the thermal annealing temperature.

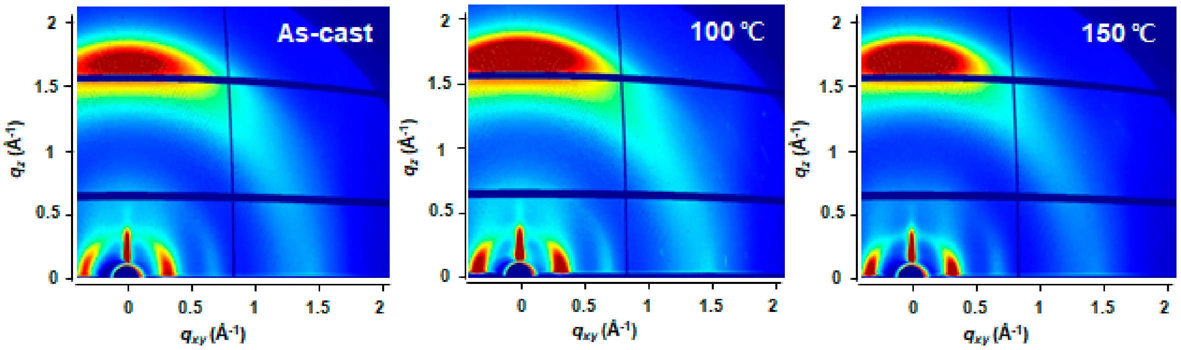

Figure S15. 2D-GIXD patterns of PBC2 depending on the thermal annealing temperature.

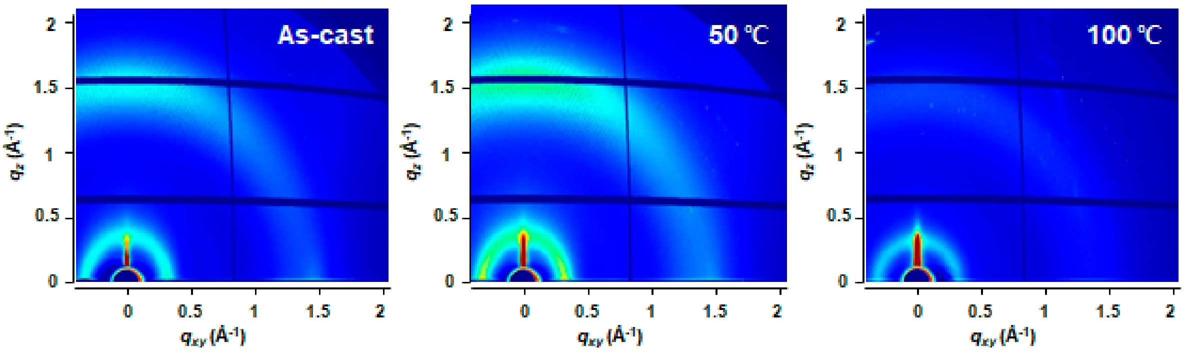

Figure S16. 2D-GIXD patterns of PBC3 depending on the thermal annealing temperature.
